# Relationship of creative projects in anatomy to medical student professionalism, test performance and stress: an exploratory study

**DOI:** 10.1186/1472-6920-9-65

**Published:** 2009-11-03

**Authors:** Johanna Shapiro, Vincent P Nguyen, Sarah Mourra, John R Boker, Marianne Ross, Trung M Thai, Robert J Leonard

**Affiliations:** 1 Department of Family Medicine, University of California Irvine Bldg 200, Rte 81, Medical Center, 101 City Dr. South, Orange, CA 92868, USA; 2 Department of Sociology, University of California, Irvine, 3151 Social Sciences Plaza, Irvine, CA 92697 USA; 3 Department of Psychiatry, Yale University School of Medicine, 300 George Street, New Haven, CT 06511, USA; 4 Office of Medical Education, Geisinger Health System, 100 N. Academic Ave, Danville, Pennsylvania 17822, USA; 5 Office of Educational Affairs, University of California Irvine School of Medicine, Berk Hall, Building 802, Irvine, CA 92697; 6 Department of Psychiatry & Human Behavior, University of California Irvine Medical Center, Neuropsychiatric Center, Bldg 3, Rte 88, 101 City Dr. South, Orange, CA 92868 USA; 7 Department of Anatomy & Neurobiology, University of California School of Medicine, Med Surg II, Room 364, Irvine, CA 92697

## Abstract

**Background:**

The anatomy course offers important opportunities to develop professionalism at an early stage in medical education. It is an academically significant course that also engenders stress in some students.

**Methods:**

Over a three-year period, 115 of 297 students completed creative projects. Thirty-four project completers and 47 non-completers consented to participate in the study. Projects were analyzed for professionalism themes using grounded theory. A subset of project completers and non-completers were interviewed to determine their views about the stress of anatomy and medical school, as well as the value of the creative projects. We also compared test performance of project completers and non-completers.

**Results:**

Projects completed early in the course often expressed ambivalence about anatomy, whereas later projects showed more gratitude and sense of awe. Project completers tended to report greater stress than noncompleters, but stated that doing projects reduced stress and caused them to develop a richer appreciation for anatomy and medicine. Project completers performed significantly lower than non-completers on the first written exam (pre-project). Differences between groups on individual exams after both the first and second creative project were nonsignificant.

**Conclusion:**

For some students, creative projects may offer a useful way of reflecting on various aspects of professionalism while helping them to manage stress.

## Background

Although the necessity and utility of anatomy lab dissection experiences have been questioned, with some schools substituting virtual training for actual cadavers, [1] the course has also been defended as the first, and crucial, training ground for aspects of professionalism, such as respect for patients, accountability for one's actions, teamwork, leadership and social responsibility, [2,3] as well as various humanistic attitudes [4,5]. In this sense, dissection is proposed as a key experience in a student's journey toward becoming a physician [6]. Anatomy is sometimes considered the student's first clinical experience, with the cadaver viewed as the student's first patient [7,8]. In addition to shaping attitudes of professionalism, academic performance in anatomy may also be an important predictor of both USMLE (US Medical Licensing Examination) Step 1 scores and passage of this critical exam [9]. Thus, the anatomy course is significant both in terms of training professional attitudes and developing academic competence.

Despite its great importance in both professionalism and academic domains, anatomy can pose a challenge for medical students. The research is equivocal about the extent to which students are bothered physically or emotionally by dissection. Several older studies report serious psychological distress in anatomy students [10-12] More recent studies, however, indicate that anatomy is only moderately, or not very, stressful [13] and in fact generates considerable enthusiasm and excitement among the large majority of students [14]. Another study indicated that students generally do not find cadaver dissection aversive, but rather see it as "a positive and challenging life event." [15] To some extent, anatomy appears to be a self-correcting experience from a stress perspective, in that student stress "naturally" appears to attenuate as the course progresses [12,16-18]. There is also evidence that learning how to manage emotions that arise during anatomy may improve test performance[19]

Anatomy lab can be the beginning of a physician's training in how to isolate and restrict their emotional responses to difficult or disturbing clinical situations, or conversely how to deal appropriately with emotions engendered by such exposure [20,21]. Unfortunately, an unintended consequence of human dissection is that it may create an inappropriate and callous "property of easiness" in dealing with death and the human body [22]. To combat this tendency, some anatomy programs have used self-reflection, [23] art, [21] journaling, [24] and group discussion [25] to encourage students to explore their emotional responses to dissection. Other curricula combine anatomy with material on death and dying [26]. This work tends to be descriptive in nature, but reports favorable reactions from students in terms of reducing stress and managing emotions.

We believe there is more to be learned about how to promote student professionalism as part of the anatomy experience; how to help students cope with the emotional aspects of dissection; and how to improve student performance in the anatomy course. To further explore these issues, we undertook the exploratory project described below. Our research questions were the following: Can completion of creative projects by students enrolled in an anatomy course: a) influence professionalism attitudes; b) improve students' self-perceived stress; and/or c) improve written and practical exam performance.

## Methods

### Subjects

Over a three year period, first year students enrolled in the anatomy course at our home institution were given the option of completing two creative projects, one after the first written exam (Time 1) and one prior to the final written and practical exams (Time 2). Students were instructed by email that they could use any artistic medium (literary, artistic, musical, performance art) to reflect on some aspect of their experience in anatomy. Faculty did not provide further direction, guidance, or advice.

Each project was worth 1.5 points toward the final course grade (out of a possible 100 points). These points were only added to the student's total point score if it made a difference in their final grade, i.e., shifting from fail to pass. During the time period studied, a total of 115 students (38.7% of all eligible students), took advantage of the creative project option. A breakdown by year and gender is provided in Table 1.

**Table 1:** Total Anatomy Students and Creative Project Completers by Year and Gender

**Year**	**# Students**	**% Female Students**	**# Completers**	**% Female Completers**
1	93	46.24	47	57.45
3	102	52.94	33	57.58
3	102	46.08	35	51.43

All students who had been enrolled in the anatomy course over a three year period (n = 93, Year 1: n = 102, Year 2: n = 102, Year 3) were sent a retrospective email, which explained the study and invited participation in the research. The study information sheet stated that creative projects would be reviewed anonymously (i.e., names and identifying information would be removed), and that all results would be reported as group data, with the exception of specific project excerpts that would only be identified by gender and year. Positive response to the email was considered to be an indication of consent, a decision approved by the institutional IRB Human Subjects committee. A total of 81 students agreed to participate in this research: 34 project completers (29.6% of all project completers) and 47 non-completers (25.8% of all noncompleting students) (Table 2). These students represented primarily a sample of convenience, i.e., students who chose to respond to the recruitment email. However, the lead researcher also personally recruited 9 students who had expressed opinions about the creative projects in an effort to ensure a variety of opinions. A subset of these 81 students, consisting of 12 project completers and 12 non-completers (total N = 24), also agreed to be interviewed about the creative projects and the anatomy course. The sample size of these interviewed students was determined by theoretical saturation of the data, i.e., interviews were discontinued when ongoing analysis of interviews began to show repetition of major constructs and themes.

**Table 2:** Study Participants by Year, Total, Project Completers/NonCompleters, and Gender

**Year**	**# Participants**	**# Completers**	**# Noncompleters**	**# Male Participants**	**# Female Participants**
1	25	12	13	12	13

2	41	17	24	12	29

3	15	5	10	6	9

### Measures/data analysis

The research team consisted of a qualitative researcher who directs the School's Program in Medical Humanities; a psychologist who is also an artist; a psychiatrist who is a poet and photographer; a second year medical student with interest in the arts; and a third year pre-medical undergraduate student. These team members analyzed all 34 creative projects. The director of the anatomy course did not participate in project analysis, but reviewed team findings for both project interpretation and student interviews at regular intervals as they emerged. Each team member brought a unique perspective to the project, while sharing an enthusiasm and some background in the arts and humanities. In team meetings, we attempted to make our various worldviews, life experience, and assumptions explicit, and to examine how these might influence our hermeneutics. The varied professional, educational, and life backgrounds of the research team contributed to our ability to transcend a single narrow disciplinary viewpoint.

The anatomy course director had retained all students' creative projects. Those projects which we received permission to include in our research were extracted and anonymized. In interpreting the themes of these projects, the research team built on an earlier analysis [27] by focusing our interest specifically on issues related to professionalism. As in our previous work, we adopted a constant comparative method based on grounded theory [28,29]. We selected this method because of the lack of existing research addressing our research questions. Qualitative research is appropriate for addressing interpretive questions, and grounded theory provides a well-established inductive theoretical framework for eliciting meaning from written texts.

Team members independently analyzed all projects by first identifying recurring words, phrases, and, and images; next, organizing these into categories; and finally into broad, overarching themes. There were regular points of comparison of among various researchers as the analysis progressed. This process allowed for the resolution of discrepancies between reviewers regarding thematic categorization and identifying each thematic code. Discrepancies were carefully inspected to ensure the authenticity of the analysis, by consulting specific examples, discussing relationships to established themes, and reaching consensus as a group. Eventually, the qualitative researcher summarized all team members' analyses. This summary was subsequently reviewed and modified by all team members in an iterative group process.

An interview schedule [27] assessed students' perceptions of anatomy-related and medical-school related stress and whether completing projects a) taught students something of value about the profession of medicine and/or b) reduced student stress. We included the interview in addition to the creative projects analyses in order to directly access the voice and perspective of students who completed creative projects and/or participated in the anatomy class. Interviews were held in a comfortable room on the medical school campus and conducted by the two student members of the research team, who were trained in interviewing techniques by the qualitative researcher. We believed that students would be more likely to be open and disclosing in the presence of interviewers whom they perceived as peers. Interviews were audio-recorded, transcribed, and anonymized. Although participants did not review study findings, at the conclusion of every interview, interviewers summarized the main points and asked for student confirmation or modification. The lead interviewer and the qualitative researcher then performed a content analysis of these interviews. Student open-ended responses to questions about stress were coded post-hoc on a 3-point scale as demonstrating either a great deal of stress (3), a moderate amount of stress (2), or little or no stress (1).

Written and practical examination scores were also collected and analyzed for all consenting students. Scores of completers versus non-completers were compared using two-tailed *t*-tests, setting the alpha criterion for significance at < .05. Gender comparisons were made using Z-tests for proportions, an appropriate alternative to chi-square tests. All computations were done using Statistical Package for the Social Sciences (SPSS) version 13.0 for Windows (SPSS Inc., Chicago, Illinois). This quantitative component was designed to address questions raised by the anatomy course director about the relationship between creative projects and student performance.

We attempted to achieve triangulation of data by examining three interrelated sources - creative projects, student interviews, and student test scores - and through utilizing a diverse disciplinary team that brought a variety of perspectives to data interpretation. All study data, including creative projects, interview tapes and transcriptions, and the research team's analytic notes, were retained in secured computer folders and locked files. This research was reviewed and approved by our university's human subjects' Institutional Review Board.

## Results

### Creative project completers

In the first year, approximately half of the class participated in the creative project option by completing at least one project; in subsequent years, approximately a third of the class participated. Female students tended to be somewhat overrepresented among those who completed creative projects (Table 1), but the difference was not statistically significant (Z = 1.58, p = .11). Of students who consented to participate in this research, the largest number came from year 2 (Table 2). Female students were significantly overrepresented (Z = 3.14, p = .003). Compared to both the study body as a whole and project completers, female students were also overrepresented in the subset of interviewed students (Z = 2.69, p = .01). The large majority of study participants who chose the creative project option completed written projects (n = 36/46; 78.3%), primarily poems (n = 19/36;57.6%) and essays (n = 13/36;39.4%) Of these, 19 were Time 1 and 17 were Time 2 projects. The art projects tended to explore the human body, relationship with the cadaver, and emotional responses to dissection in ways that mirrored the written projects.

### Professionalism

We identified three major themes in our analysis of all projects, each of which was in some way related to professionalism issues. We labeled the first theme **Reflections on Doctoring**. Overall, two-thirds of students' projects addressed in some form how dissection taught them not only anatomy, but also the importance of teamwork, good role-modeling, and a deepening understanding that medicine is a humane calling rather than a technical career.

"I feel that I have undergone tremendous personal growth, becoming a more sensitive, socially conscious individual. Whereas before I use to jump at the opportunity to view surgeries, procedures, and conditions, in a sense not thinking about the person as a whole, but viewing the human body as a marvelous machine; now I get a bit uneasy, I began to reflect on the individual who is undergoing the procedure, their loved ones and how they all must feel. Now I usually say a little prayer for them, take a deep breath and do my best to serve them in any way possible." (#29, female, year 2)

A little over one-quarter of students specifically explored what it meant to become a doctor, emphasizing that knowledge acquisition was insufficient, and the importance of qualities of respect, humility, caring and appreciation.

The second theme we identified was **Relationship with the Cadaver**. Almost half the students reflected on some aspect of the student-cadaver relationship. Slightly more than half of the students considered the cadaver as a tool for learning or imagined the former life of the cadaver. A smaller number portrayed the cadaver as their teacher, often from the perspective of the cadaver.

"But don't you cringe or feel

Any sorrow for my death or life.

Just focus and dissemble this shell

With your quaking, gleaming knife.

And be not afraid, for the most grotesque

Offense you would commit

Is to drop the line and jump the deck

Before you knew all of it." (#41, male, year 1)

Only a handful thought of the cadaver as their first patient.

The final theme we found was the student's **Emotional Response to Dissection**. The most frequently expressed emotion (by about half the students) was gratitude and thankfulness toward the cadavers for the donation of their bodies. Over a third of the students expressed awe at the wonders of the human body. However, about a third of students also expressed or portrayed concern regarding their actions toward the cadavers, especially a sense of becoming emotionally detached and desensitized.

"Old man, I know not your name

And nor who you are

But don't you explain!

I need nothing but these

Damned utensils, so

Please,

Do not try to move me

To thoughts of your life,

Who you might be

Or rather, wherefore

I just can't care" (#41, male, year 1)

Almost one-quarter of students referenced negative emotions of shame/guilt, fear/anxiety, and sense of sacrilege.

### Shifts in professionalism: *reflections on doctoring*

Student projects were more likely to reflect on what anatomy had to teach them, and what the practice of medicine entailed, at Time 1 vs. Time 2, although large numbers at both periods acknowledged an alteration in their views (75% of students at Time 1, almost 60% at Time 2). Approximately equal percentages of students at Times 1 and 2 explored what becoming a doctor involved. Students at Time 2 were almost three times as likely to engage in specific personal reflection about how they were changing as a result of their medical training.

### Shifts in professionalism: *relationship with cadaver*

This was a topic of significance to respondents at both time periods, but students tended to address this issue in their projects somewhat more frequently in the final project. Students at Time 1 were much more likely to speculate about the person of the cadaver:

"There were times during lab when I would find myself, unconsciously, rubbing our 'patient's' arm, wondering how she died and whether or not she knows what is happening to her body right now." (#99, female, year 2)

Students at Time 1 were the only ones to talk about the cadaver as their first patient. Conversely, students at Time 2 were much more likely to mention the cadaver as a means of advancing their own learning. Approximately equal percentages of students at Times 1 and 2 regarded their cadaver as a teacher, although Time 2 students were slightly more likely to do so.

#### Shifts in professionalism: *Emotional responses to dissection*

While gratitude was by far the most common emotions shared by students, negative emotions were prevalent at both Times 1 and 2, with between a fifth and a quarter of students expressing guilt, shame, fear, or anxiety.

"I must admit that the thought of taking human anatomy was a scary one. Despite taking a Human Anatomy Lab course as an undergraduate five years ago, I felt nervous ..." (#29, female, year 2)

However, a sense of sacrilege was in evidence much more frequently at Time 1:

"And when my lab partner cut into her breast, it made me angry. How dare he? The act felt violent and violating." (#22, female, year 3)

Overall concern about dissection was also judged to appear more often in initial projects. By contrast, students' joy at the revelations resulting from dissection almost doubled from Time 1 to Time 2, as did students' sense of awe at the mysteries of the human body. Students' early worries about emotional distancing were largely replaced by an attempt to achieve emotional balance:

" [I am] attempting to distance myself in a professional manner while maintaining some empathy" (#52, female, year 2)

Although the emotion of gratitude was widespread at both time points, it did increase noticeably in the final projects.

"Learning, admiring/Just feeling honored and privileged to learn/

From selfless and pale phantom teachers" (#59, female, year 3)

Overall, dissection was generally viewed as less sacrilegious and more acceptable by the end of the year. Students expressed self-pride and enthusiasm for having completed a great adventure, and imagined a satisfied cadaver:

" [I have] no regrets or reservations...

My purpose fulfilled/

The ability to teach and heal/...

Lives granted strength and happiness." (#24, male, year 2)

### Evolving professionalism based on student interviews

Ten of 12 completers felt completing a creative project improved their self-awareness and ability to reflect on experience.

"I think it has taught me like, um, [to] think [about] what you feel at the moment because usually with the whole rush of med school, you're just going through the motions, and doing something like this, such as the project, gives you a chance to, um, to kind of reflect on [feelings] that you wouldn't have before." (#21, female, MS2)

The majority of students felt completing the projects also changed or reinforced their attitude toward medicine in a positive way:

"I think if the project wasn't there, I would be more inclined to think that the profession was more brutal and not very humanistic at all." (#18, female, MS1)

A smaller number felt the project gave them additional insight into the doctor-patient relationship. Students who reported this response tended to detect a continuum from care of the cadaver to patient care. These students mentioned the importance of having respect for the patient, developing emotional connection with the patient, having empathy for the patient's subjective experience, and being able to interpret both the medical and emotional *meaning *of a patient's symptoms.

"The project helped [me] connect the whole anatomy class to real people. These are things that you see on real people and they will be on your patients, on yourself, and so that's a tie to real life..." (#2, female, MS1)

Several students reported that doing the project improved their attitude toward anatomy in particular. One person who expressed a positive valuation noted that the project helped instill an attitude of wonder and respect for the cadavers. Another student commented that doing the project gave her a boost during dissection by making her more interested in body parts that she could incorporate into her creative project. Still others commented that they learned to think about the perspective of the cadaver. Even non-completing students seemed to appreciate the ability of the projects to stimulate self-reflection:

"I loved seeing my classmates' artworks displayed, and I liked the fact that it made me [think], well, how do I express my anatomy experiences? And I have thought about it, how is anatomy affecting me? How [could] I express it?" (#22, female, MS1)

### Relation of stress to project completion

Most project completers (9/12) thought anatomy was either stressful or very stressful. Fewer non-completing students rated anatomy as stressful or very stressful (5/12). (The remaining students in both groups described anatomy as producing little or no stress). The primary sources of stress for both groups appeared to be information overload.

"I felt I was always studying for anatomy. I enjoyed the material, but it was a constant stressor b/c of the amount of work it took to excel in the course"; "There's so much material and you can't learn it all." (#e1, female, MS2)

Several completers also noted lab-related stress, describing it as gross, disgusting, and generally uncomfortable. Female students reported higher levels of stress than males, but these were non-significant except for the stress of practical exams (p = .04).

Most completers (9) felt that participating in the creative project option reduced either anatomy-related stress, or medical school stress in general. They saw the project as a relaxing break, a way to avoid burn-out, a calming, tension-releasing experience, and a means of attaining a different, and healthier, perspective on anatomy.

"I think it was more a way for me to take the class and not go crazy ... I think it [the project] kept my interest, so like I said, not getting burned-out..." (#2, female, MS1)

Completing creative projects provided an outlet for difficult emotions, offered a way to acknowledge the "feeling" aspect of medicine, to explore multiple perspectives, and through reflection, to resolve contradictory response to the anatomy experience. About half of the non-completing students spontaneously made positive comments about the value of the creative projects to reduce their own stress.

" [Seeing] the anatomy projects like helped me get through some of the things I was dealing with." (#8, male, MS2)

### Test performance

Project completers performed significantly less well than noncompleters on the first written exam; and the superior performance of noncompleters approached but did not achieve significance on the first practical exam (both of these exams occurred pre-project completion). After completion of the first creative project, there were no significant differences on the subsequent individual exams; and this pattern continued to hold after the second creative project was completed. However, project non-completers performed better than completers on all exams. When all exam scores were combined and analyzed as a single score, the mean difference between non-completers and completers achieved statistical significance (Table 3).

**Table 3:** Exam Scores/Final Grades by Project Completers/Non-Completers

	**Completing Creative Projects?**	**N**	**Mean**	**S.D**.	t-value**	**d.f**.	**p value**
Midterm 1 (Written)	No	42	86.72	6.81	1.98	78	0.051
				
	Yes	38	83.21	8.97			

Midterm 2 (Practical)	No	42	87.94	6.47	1.80	78	0.08
				
	Yes	38	85.37	6.28			

Midterm 2 (Written)	No	42	82.71	7.49	1.37	78	0.17
				
	Yes	38	80.25	8.55			

Midterm 3 (Practical)	No	42	84.62	7.23	1.65	78	0.10
				
	Yes	38	82.05	6.62			

Midterm 3 (Written)	No	42	83.44	8.62	1.35	78	0.18
				
	Yes	38	80.87	8.43			

Final (Practical)	No	42	87.52	6.70	0.92	78	0.36
				
	Yes	38	86.15	6.63			

Final (Written)	No	42	87.32	7.13	1.87	78	0.07
				
	Yes	38	84.19	7.83			

Combined Test Scores	No	42	85.75	5.34	1.95	78	0.05
				
	Yes	38	83.16	5.54			

*First creative project submitted before midterm 2; second creative project submitted before final exams

**Independent 2-tailed t-test assuming equal group variances

### Relation of project completion to test performance

Most project completers did not believe that doing creative projects enhanced their knowledge acquisition:

"I don't think that I learned anything about anatomy specifically... learning this nerve goes here, here, and here, the project, I don't think it aided in that at all."(#15, male, MS2)

However, one or two students each mentioned that, as a result of the project, they felt they were more attentive, could pay better attention to details, were more curious about anatomy, were more comfortable with dissection, and had greater "ownership" of the course in the sense that they were more actively engaged with the material, and from a greater range of perspectives (i.e., anatomical, biomedical, aesthetic, and emotional).

"...The project really gave me something else to be looking at while I was dissecting... so just having that little 'ump' I'm on the search for cool things in the body... helped my anatomy experiences a lot... I'm hoping that I'm gonna keep this curiosity and just...um... and looking for the spectacular...um...special things in medicine other than just focusing uh... here's what you do." (#2, female, MS1)

## Discussion

Both according to project thematic analysis and student interviews, completion of creative projects improved attitudes of professionalism by promoting self-reflection about anatomy and doctoring, examining the student-cadaver relationship, and exploring emotional responses to dissection. These findings accord with Lachman and Pawlina's assertion of the value of integrating reflective practices into the anatomy curriculum as a method for promoting professional attitudes [29]. Students at both time points remained actively engaged in reflecting on the meaning and significance of anatomy in particular and medicine in general. Later projects were more likely to reflect on the effects of the anatomy course on students personally. Overall, Time 2 projects showed more emotional balance, but also a greater tendency to see cadavers as means to the end of students' own education; initial projects tended toward emotional over-identification with cadavers, but were also more likely to imagine them humanistically. Final projects were more likely to demonstrate greater resolution and comfort with the necessity of dissection; articulate awe and wonder at the intricacies of the human body; and express appreciation for the gift of the donor, rationalizations similar to those reported in previous work [30]. Student interviews confirmed that completion of creative projects improved attitudes toward both the anatomy course and the profession of medicine. In particular, students reported increased self-awareness and ability to engage in reflection as benefits developed by the projects. Even students who did not do the projects generally reported positive effects from viewing the work of their peers.

Students who chose to complete creative projects were more likely to report experiencing anatomy as moderately to very stressful than were noncompleters, although their perceptions about the stressful nature of medical school in general were similar. Both completers and noncompleters attributed the majority of their stress to the quantity and complexity of the information to be learned. Interestingly, although creative projects did not address information overload and in fact may have taken away from study time, project completers still found the projects to be a useful way of reducing stress. Because they may have been more anxious about their test scores, and looking for ways to bolster their grades, the additional extra credit points offered may also have served as an effective stress-reducer.

In terms of test scores, project completers performed significantly less well on the first (pre-project) written exam. This finding must be interpreted with caution because of the small numbers of students who participated in this research (27.3% of eligible students overall). It is possible that, with a better response rate, this finding might not have been sustained. However, even if it proved to be a reliable difference, we do not know why it occurred. Completing students, who also reported themselves being more stressed by anatomy, might have been more anxious about the exam, and therefore performed less well. As a group, they might also have been poorer academic performers. Completers' scores on written exams narrowed the gap with non-completers after both the first and second creative projects, but it is not possible to attribute a cause and effect relationship. Many other factors may have influenced this small improvement, including additional exposure to course material, increased studying/academic effort, and more comfort with medical school in general. It is possible that, by reducing stress, the projects indirectly enabled completers to improve test performance somewhat.

Female students were both more likely to select the creative project option and to participate in the research study than were male students. The overrepresentation of women choosing creative projects was not significant; but their overrepresentation in the actual research (including both project completers and noncompleters) *was*. It is possible that while women *may *have a slightly greater tendency to become involved in the creative, "right-brain" side of medicine; they may have a significantly greater tendency than men to engage in an altruistic behavior such as participating in a research project where they derive no direct benefit. Considering this difference in the context of a recent finding that female medical students score consistently higher on measures of empathy than do male students [31] suggests the need to find ways of encouraging both altruism and its close relation empathy in male students, as essential qualities in clinical practice.

The findings of this exploratory project must be interpreted cautiously within the limitations of the study. This study was conducted at a single medical school. Further, because of the retrospective design, only a little over one-quarter of eligible students chose to participate in the research; and our sample was skewed toward female respondents, who seemed more willing to help out the researchers. Other than gender and age (which was nonsignificant), we did not have access to any comparative data for responders and nonresponders. However, we believe that the most likely explanation for the low response rate was simply students' sporadic reading of their email (an often-complained-about phenomenon at our medical school), as well as students' reluctance to make additional efforts, no matter how minimal, beyond their coursework and ongoing extracurricular commitments. In support of this hypothesis, we did not receive *any *emails from students *declining *to participate in the research. A further potential limitation is that, because extra credit points were offered to students who completed projects, there may have been a bias introduced regarding the character or motivation of students who pursued this offer. However, although students acknowledged the influence of the extra points, they were able to expatiate convincingly on the more intangible benefits derived from their participation in the creative projects. Finally, since this was not a comparative study of different methods of intervention, we cannot comment on the relative merits of using creative projects versus other strategies mentioned in the introduction to promote reflection or reduce stress.

## Conclusion

Despite its limitations, we believe our report offers intriguing preliminary information on several dimensions. First, this study suggests that creative projects are an effective way to encourage self-examination about the process of doctoring and the student-cadaver (patient) relationship. It also indicates that such projects may be a way of helping students who experience anxiety and stress to first explore, and then reduce, these negative emotions. It was unclear whether completion of creative projects had a significant impact on test performance per se, but this possibility deserves further investigation. Overall, integrating creative projects in anatomy courses should be considered as a simple method of enhancing and enriching this important introduction to doctoring.

## Competing interests

The authors declare that they have no competing interests.

## Authors' contributions

JS was the project director. She developed and helped to implement the creative projects option for the Anatomy course. She oversaw all data collection, developed the student interview schedule, and trained the two interviewers. She also led both the qualitative and quantitative data analyses, including facilitating the constant comparative method through research team emails and meetings. She had primary responsibility for writing the final article. VPN performed the literature review; organized the data; conducted all medical student interviews; quantified student stress scores; and participated in the qualitative analysis of the student projects. SM participated in the medical student interviews; and participated in the qualitative analysis of the student projects, contributing her perspective as a medical student. JRB provided advice about test score data, stress assessment, and performed all statistical analyses. MAR participated in the qualitative analysis of the student projects, contributing her perspective as a clinical psychologist with expertise in medical student wellbeing; and an artist. TMT participated in the qualitative analysis of the student projects from his perspective as a psychiatrist, poet, and artist. JRL approved, supported, and the introduction of the creative projects option in the Anatomy course. He offered insights about data interpretation based on his role as course director of anatomy. All authors reviewed and approved the final article.

## Notes on authors

Johanna Shapiro, Ph.D. is Professor of Family Medicine and Director of the Program in Medical Humanities at UC Irvine School of Medicine.

Vincent Nguyen graduated from UC Irvine with a B.S. in biological sciences and a B.A. in sociology. He is currently applying to medical school.

Sarah Mourra, M.D. graduated with distinction in medical humanities from UC Irvine School of Medicine; and is currently a resident in the Department of Psychiatry at Yale University School of Medicine.

John Boker, Ph.D. is vice-president of faculty/curriculum development at Geisinger Health System.

Marianne Ross, Ph.D. is a clinical psychologist and artist who works with the UC Irvine School of Medicine Office of Medical Education.

Trung Thai, M.D. is an associate professor of psychiatry at UC Irvine School of Medicine, as well as a poet and photographer.

Robert J. Leonard, Ph.D. is an Adjunct Professor of Anatomy and Neurobiology, the Anatomy and Embryology Course Director at UC Irvine School of Medicine, and author of a highly regarded anatomy textbook.

## Pre-publication history

The pre-publication history for this paper can be accessed here:


